# Navigating Masculinities: An Exploration of Peer Coaching, Health Behaviors, and Mortality Risks Among Men in Tuberculosis and HIV Interventions in South Africa

**DOI:** 10.1177/15579883261430886

**Published:** 2026-03-13

**Authors:** Kelly Rutt, Lindsey DeVos, Kuyola Gala, Thembani Njama, Mziwanbantu Fosi, Zibele Mhaleni, Joseph Daniels, Andrew Medina-Marino

**Affiliations:** 1 Division of Infectious Diseases, David Geffen School of Medicine, University of California, Los Angeles, Los Angeles, CA, USA; 2 Desmond Tutu HIV Centre, University of Cape Town, Cape Town, South Africa; 3 Edson College of Nursing and Health Innovation, Arizona State University, Tempe, AZ, USA; 4 Department of Psychiatry, Perelman School of Medicine, University of Pennsylvania, Philadelphia, PA, USA

**Keywords:** masculinity, peer coaching, men’s health, tuberculosis, HIV retention

## Abstract

South African men remain disproportionately affected by tuberculosis (TB) and HIV, with social norms around masculinity often hindering care-seeking and medication adherence. This study investigates how Coach Mpilo, a peer mentoring intervention led by men who are TB survivors or living openly with HIV, engages concepts of masculinity to improve health outcomes among men in TB/HIV care. We conducted in-depth qualitative interviews with male peer coaches implementing the Coach Mpilo intervention in Eastern Cape, Western Cape, and KwaZulu-Natal provinces. Coaches, all TB survivors and/or living with HIV, described their experiences supporting newly diagnosed men with TB/HIV. Guided by the Health, Illness, Men, and Masculinities Framework, thematic analysis identified key domains through which masculine norms and social expectations influenced health trajectories and intervention engagement. 20 participants described how men’s emotional health is constrained by norms discouraging vulnerability. Participants reported that men’s self-reliance and clinic avoidance impede timely care and adherence, and how provider roles, poverty, and structural barriers increase risk, often forcing men to choose work over health care. Coaches discussed hegemonic ideals among their peer clients, and that stigma delays diagnosis and worsens outcomes. Coaches described finding success by framing treatment and health-seeking as acts of strength and responsibility, and by advocating for male-centered clinic spaces and tailored support. Findings illustrate that working with, rather than against, masculine norms can motivate men to engage with TB/HIV services. Peer coaching that reimagines vulnerability as strength, while addressing both social and systemic barriers, holds promise for promoting men’s health and reducing disparities in high-burden settings.

## Introduction

Men’s health represents one of the most pressing yet under-addressed global public health challenges. Globally, male life expectancy is substantially lower than that of females, with men living an average of ~5 years less (Baum et al., 2021; Thorpe et al., 2016). This disparity is particularly pronounced in specific health domains, including cardiovascular disease, lung cancer, alcohol-related liver disease, accidents, and suicide (Ji et al., 2023; Lababidi et al., 2023; Patwardhan et al., 2024; Sorenson, 2011; Tan et al., 2023). The disparity in morbidity and mortality from lower respiratory infections, including COVID-19 and Tuberculosis (TB), is also significantly greater among men compared to women, with TB being the single largest infectious cause of death in human history (Patwardhan et al., 2024; World Health Organization, 2024). Despite these well-documented disparities, men’s health remains largely absent from global public health policy initiatives, with health organizations and governments continually failing to address men’s unique needs as a distinct priority requiring targeted interventions (Griffith, 2024).

In South Africa, men face a complex intersection of health challenges and inequities that exemplify global patterns while reflecting unique contextual factors. These disparities are driven, in part, by sociostructural barriers that result in poorer treatment outcomes across multiple health conditions, greater workplace, community, and marital prospect-related stigma, men’s higher rates of risk-taking behaviors (e.g., condomless sex, binge drinking, smoking), and suboptimal health-seeking behaviors (Beia et al., 2021; Krishan et al., 2014; Kubjane et al., 2023; Motlhale et al., 2022; Moyo et al., 2023; Sharma et al., 2017; Tsai & Siedner, 2015; Zuma et al., 2022). Gender disparities in TB are especially pronounced in South Africa, where men account for over 60% of people with microbiologically confirmed TB, are less likely to access testing, and experience a disproportionate burden of poorer outcomes (Kubjane et al., 2023; Mlotshwa et al., 2016; South African National Department of Health, 2025). Pulmonary TB is responsible for 81% of the 11.2-year sex difference in life expectancy among those with HIV and 43% of the 13.1-year difference among those without HIV (Reniers et al., 2017). While a growing body of literature suggests sex-specific factors and the differential susceptibility of males and females to TB disease, socio-behavioral factors reflect broader challenges in how social and gender norms shape health-seeking behaviors, treatment adherence, and engagement with health care systems (Hertz & Schneider, 2019; Nhamoyebonde & Leslie, 2014).

Barriers to health care engagement are often created by the coaction of hierarchical masculinities, including hegemonic ideals of stoicism and self-reliance, interacting with broader structural inequities (Evans et al., 2011; Rwafa-Ponela et al., 2022; Stern, 2015). Understanding these dynamics is essential for developing effective interventions that address both individual behaviors and systemic factors contributing to poor health outcomes among men. Masculinity and gender norms significantly influence men’s TB and HIV treatment outcomes (Chikovore et al., 2015; Chinouya & Adeyanju, 2017; Daniels et al., 2021; Medina-Marino et al., 2019, 2022; Siu et al., 2014). These norms prioritize men’s provider roles and associate illness with weakness (Chikovore et al., 2020; Horton et al., 2016) while stigma reinforces these norms (Krishan et al., 2014). Consequently, men fear disclosing their status and avoid accessing care, isolating them from necessary support systems (Chikovore et al., 2020; Daftary & Padayatchi, 2012; Medina-Marino et al., 2022; Sharma et al., 2017).

In qualitative interviews exploring men’s social networks, TB testing behavior, and treatment experiences, men explicitly stated their desire for peer-to-peer support to navigate TB-related stigma and unhealthy masculinity norms (Medina-Marino et al., 2022). These findings aligned with broader evidence demonstrating that peer-based interventions facilitate meaningful changes in health-related behavior, including treatment adherence and health care engagement, with small- to medium-sized effects across diverse health outcomes (Berg et al., 2021; Øgård-Repål et al., 2023). Peer coaching addresses several key barriers that disproportionately affect men’s engagement in TB care, including the perception that help-seeking threatens masculine identity, fear of judgment from health care providers, and discomfort with clinic environments experienced as feminized spaces (Medina-Marino et al., 2022). The peer coaching mechanism is particularly well-suited to these challenges. By having TB survivors and men living openly with HIV serve as coaches, the intervention creates relational spaces where vulnerability can be reframed as strength and treatment adherence can be positioned as an act of masculine responsibility rather than weakness. Critically, in our formative work, men advocated not only for peer support but also for interventions delivered in familiar locations where men congregate (Medina-Marino et al., 2019). Men have expressed interest in peer-to-peer support interventions in “men’s spaces” to discuss shared experiences, build skills to navigate stigma, and identify resources for treatment completion (Daniels et al., 2017, 2021; Siu et al., 2013). This underscores that patient-centered preferences must guide intervention design and prioritizes men’s lived experiences and stated needs, ensuring the intervention resonates with its target population.

In this study, we sought to explore how masculinities shape health outcomes among men, and how peer-support interventions can improve men’s overall well-being and retention in TB and TB/HIV treatment. These explorations were conducted in the context of the Coach Mpilo program, a peer-support intervention to engage and retain men in TB and HIV care and treatment services in South Africa (Bruns, 2021; Hlongwa et al., 2022).

## Method

### Study Overview and Setting

This qualitative study employed in-depth interviews with men implementing the Coach Mpilo program, also known as Coach Mpilo Coaches (i.e., Coaches). Coach Mpilo is a peer-support intervention, delivered by coaches who are TB survivors and/or living openly with HIV, to engage and support men (players) newly diagnosed with TB and/or HIV or those struggling with treatment adherence. Coaches interviewed for this study were either: (a) programmatically implementing the Coach Mpilo program for men living with HIV via PEPFAR-funded partner organizations in Western Cape and KwaZulu-Natal provinces, or (b) nested within a larger mixed-methods study (NIH R34HL170819) examining the feasibility of a newly adapted Coach Mpilo intervention for men newly diagnosed with TB or TB/HIV in Eastern Cape province. In 2023, the estimated proportions of men aged ≥25 years living with HIV in KwaZulu-Natal, Eastern Cape, and Western Cape were 21.6%, 27.5% and 11.3%, respectively (Johnson & Dorrington, 2024; Johnson et al., 2017). That same year, the Eastern Cape had the highest TB notification rate (703 per 100,000 population), followed by the Western Cape (~500–599 per 100,000 population) and KwaZulu-Natal (~400–499 per 100,000 population) (South African National Department of Health, 2025).

### Participant Recruitment and Data Collection

We recruited coaches participating in the Coach Mpilo TB and TB/HIV feasibility study. In addition, PEPFAR partner organizations implementing the Coach Mpilo program were contacted by the study team to seek their engagement and support for the study. Interested organizations facilitated introductions to their coaches, who were willing to be interviewed. Men who had completed Coach Mpilo training, were active coaches within the past 3 years, and were aged 18 years or older were eligible to participate. Although the original plan was to conduct up to 40 interviews, PEPFAR and USAID funding cuts in early 2025 resulted in the termination of numerous HIV/TB programs across South Africa, which included many Coach Mpilo positions (Ndjeka et al., 2025). This action limited our final sample to 20 completed interviews. Despite this, we achieved theoretical saturation after approximately 15 interviews, with no new substantive themes emerging in subsequent interviews. This aligns with established qualitative research methodologies, indicating that 9–17 interviews are typically sufficient to reach saturation for studies with homogeneous populations and narrowly defined objectives (Guest et al., 2020).

Semi-structured interviews, conducted in participants’ preferred language, took place in private settings, ensuring participant confidentiality and comfort. Interviews lasted approximately 60 minutes. The interview guide was designed to elicit detailed narratives about coaches’ experiences with players, their strategies for addressing masculine norms, and the challenges they face in promoting health behaviors. Specific questions explored how coaches navigated conversations about traditional masculinity with their players, the techniques used to encourage health-seeking behaviors, and observations regarding the influence of masculine identities on treatment adherence. All interviews were conducted by isiXhosa-speaking South African men with lived experience of the cultural context and sensitivities associated with discussing masculinity and health with other men. Interviews were audio-recorded, transcribed verbatim, translated from isiXhosa into English (if necessary), and then reviewed by a second study team member for quality and accuracy.

### Data Analysis

Interview transcripts were read by the study team, during which thematic domains emerged organically through memoing with a contextualist epistemological stance, processing participants’ accounts while interpreting them in relation to cultural and gendered norms. We employed a codebook approach to thematic analysis, using an iterative process that combined inductive identification of themes from the data with deductive organization guided by the Health, Illness, Men, and Masculinities (HIMM) framework. The team paid specific attention to how masculinity influenced men’s health. These domains were defined by identifying patterns and descriptions of behaviors reflecting the impact of masculinities on health outcomes, with theory-informed organization of themes using the HIMM constructs as a scaffold. An initial codebook was constructed iteratively based on memos and subsequent readings of a subset of the transcripts. Themes initially emerged inductively from participants’ narratives, which were then organized deductively into HIMM’s conceptual domains, while remaining open to emergent concepts that spanned multiple domains. The remaining transcripts were coded using this draft codebook, with additional quote samples identified to further refine themes, in Dedoose (Version 9.0.17, Los Angeles, CA: SocioCultural Research Consultants, LLC). To ensure reliability and trustworthiness, transcripts were coded independently by two research team members, with discrepancies resolved through consensus discussions and community-checking. The analysis process included familiarization with the data, iterative memoing, systematic coding, and the ongoing development and refinement of themes. The resulting themes were organized according to the four key conceptual domains, while also accommodating emergent concepts spanning multiple domains. This approach enabled a comprehensive analysis of how masculinities shape health behaviors and outcomes among men participating in TB/HIV interventions (Table 1).

**Table 1.** Domains of Masculinities and Health: Definitions and Distinctions.

Domain	Definition	HIMM construct(s) engaged
Masculinities and Well-Being	Examines how masculine norms and expectations affect men’s emotional health, social support, and overall well-being, influencing their ability to seek help, process emotions, and adhere to treatment.	Hierarchical masculinities (stoicism), structural constraints on support
Masculinities and Health Behavior	Explores the ways masculine ideals shape men’s health-related actions, including health-seeking, clinic attendance, risk-taking, and adherence to medication, often acting as barriers or motivators for engaging with care.	Hegemonic norms + structural clinic barriers
Masculinities and Physical Health	Focuses on the direct impact of masculine norms on men’s physical health, including symptom recognition, illness denial, and management of chronic conditions, as well as how structural factors (e.g., poverty, food insecurity, job insecurity) intersect with gender roles.	Masculine strength/denial; life course (e.g., younger vs older ideals)
Masculinities and Mortality Risk	Investigates how adherence to traditional masculine values contributes to late diagnosis, advanced disease progression, and increased risk of premature death, and how interventions can disrupt these harmful patterns.	Structural inequities + harmful masculine scripts

### Theoretical Framework

The HIMM framework was used as a critical lens for analyzing how masculinities intersect with social determinants to shape men’s health outcomes (Evans et al., 2011). Grounded in Connell’s theory of multiple masculinities, HIMM positions gender as a dynamic social determinant that interacts with race, class, sexuality, and other structural factors across the life course (Evans et al., 2011; Scott, 2015). This framework emphasizes three key dimensions: (a) life course trajectories, where masculinities evolve through youth, middle age, and later life, influencing health behaviors like risk-taking or care avoidance; (b) hierarchical masculinities, including hegemonic ideals (e.g., stoicism), complicit adherence, and marginalized identities; and (c) structural inequities, where systemic barriers (e.g., poverty, stigma) exacerbate health disparities (Courtenay, 2000; Evans et al., 2011). HIMM challenges individualistic health models by centering how men’s engagement with health care is mediated by masculine norms and institutional contexts, offering a scaffold for interventions that address both personal and systemic drivers of health choices.

### Ethics and Positionality

Ethics approval was provided by the University of Cape Town Human Research Ethics Committee (Ref no.: 523/2023) and the Institutional Review Board of Arizona State University (Ref no.: STUDY00019001). The study was implemented with the permission and approval of the provincial health departments in which coaches implemented the Coach Mpilo program. All participants provided written informed consent and were reimbursed with R150 (approximately 7.5 USD) for their time.

Our research team brought diverse positionalities to this study that shaped data collection and interpretation. All interviews were conducted by isiXhosa-speaking South African men with lived experience of the cultural context, which facilitated culturally grounded conversations about masculinity and health and brought insider perspectives that informed our understanding of how masculine norms operate in participants’ lives. The first author is a white woman from the United States, and the principal investigator is a white man from the United States, positions that brought both distance and the need for careful attention to cultural humility throughout the analysis. Given that participants were intervention staff implementing Coach Mpilo, there was potential for social desirability bias in their responses. However, the fact that interviewers shared cultural and linguistic backgrounds with participants likely facilitated more candid discussions about challenges and failures, as well as successes. We remained attentive throughout analysis to how our varied positions, as insiders and outsiders, researchers and practitioners, men and women, shaped our interpretation.

Quotes are attributed to participants using the Participant ID Number, the Coach Mpilo program they were implementing (HIV only or TB/HIV), the length of time they had been implementing the Coach Mpilo program, and their provincial location.

## Results

Given the design and purpose of the Coach Mpilo program, all coaches were men (Table 2). The median age of coaches interviewed was X (IQR), and ranged in age from 24 to 55 years. Most were implementing the Coach Mpilo program in the Eastern Cape, and all but three were coaches within PEPFAR partner organizations. Nearly all coaches interviewed (95%) were living with HIV, with the overwhelming majority also being TB survivors; the only coach not living with HIV was implementing the feasibility research study that included players only diagnosed with TB without HIV co-infection.

**Table 2.** Socio-Demographic Characteristics of Coach Participants.

Characteristic	*N* = 20 (n; %)
**Age** (years)	
Median (IQR)	
Range	24–55
**Gender**	
Male	20 (100.0%)
**Marital Status**	
Single	11 (55.0%)
Married/Living with partner	6 (30.0%)
Separated/Divorced/Widowed	3 (15.0%)
**Provincial Location**	
Eastern Cape	14 (70.0%)
Western Cape	5 (25.0%)
KwaZulu-Natal (KZN)	1 (5.0%)
**Coach Program Type**	
PEPFAR Program Coach Mpilo (HIV Only)	17 (85.0%)
Research Study Coach Mpilo (TB or TB/HIV)	3 (15.0%)
**Years as Coach**	
<1 year	4 (20.0%)
1–3 years	6 (30.0%)
4–5 years	9 (45.0%)
>5 years	1 (5.0%)
**Living with HIV**	19 (95%)
**History of TB**	15 (75%)

In-depth interviews revealed four key thematic domains through which social expectations of manhood influence the health trajectories of participants undergoing TB and/or HIV treatment in South Africa. They offered unique insights into how masculine norms influence health behaviors and treatment adherence among their players. Their narratives, collected across three geographic regions, illuminate the complex interplay between hierarchical masculinities, structural inequities, and health outcomes.

### Masculinities and Well-Being

The data uncovers how norms of emotional restraint and self-reliance leave many men managing TB/HIV with limited avenues to process distress, which in turn undermines adherence and ongoing engagement in care.

#### Emotional Isolation as a Barrier to Care

Coaches reported that many players arrived already carrying unresolved trauma, strained family relationships, or economic stress, yet felt unable to talk about these issues with relatives or peers. They often discussed how the inability of players to process emotions and access support networks had direct consequences for their treatment adherence and overall well-being. Many coaches observed that players who remained emotionally isolated were more likely to struggle with accepting their diagnosis, adhering to treatment, and remaining engaged in care. As one coach explained, men “cannot share anything with anyone, so they keep things inside . . . that gets in the way of their adherence and treatment collection” (001, TB/HIV, 11 months, Eastern Cape).

*There was a moment, where a person would admit in front of you and say that they accept coaching, I will walk with you but when you try to call him, he does not pick up . . . only to find out that when you do a follow up . . . you find that he is a very private person who does not like to talk, he does his own thing, he knows his story and he does not need any assistance from anyone in his life . . . There are those who are not doing well, whereby a person will start and then he runs away and does not come to the clinic, you trace him and go look for him at his home, see you and say “sure Bro! I am coming” then* [he made a sound that is used when someone runs away]. (017, HIV, 5 years, Eastern Cape)

Emotional withdrawal was not only an immediate reaction to diagnosis but an ongoing stance that made it harder for men to accept their HIV/TB status, sustain daily treatment, or return to the clinic when they felt judged or unsupported at home. Conversely, when men are provided with supportive environments that validate their emotional experiences and encourage open communication, they demonstrate improved mental health outcomes, stronger treatment adherence, and enhanced ability to navigate the challenges of living with chronic illness.

*The player was now comfortable, and you could see that he now understood his situation, and he was now able to open up, because I was the first person he met when he first came to the clinic* [first diagnosed]. *He was comfortable because of my coaching style as I have explained before on how I work with players*. (005, HIV, 2 years, Eastern Cape)

#### Peer Disclosure as a Bridge to Vulnerability

Coaches consistently emphasized emotional disclosure as a powerful counterforce to traditional masculine norms of stoicism. Sharing their personal experiences of living with HIV and/or being a TB survivor builds players’ trust and openness necessary for sustained treatment adherence. Coaches observed that men often experience hurdles to treatment collection and clinic attendance, while those who receive emotional support through peer coaching demonstrate improved well-being and stronger treatment adherence.

*I would say it is sharing my own story with them. I see that as a key and something that works for me. I, as soon as I meet a player, I always share my own story. I don’t want to appear as a professional to them or as a nurse. I share my story and tell my player I know TB from my own experience, I once had it.*(002, TB/HIV, 11 months, Eastern Cape)

*So, when you hear something from someone who has learnt about it and [vs] someone who has walked the journey, you can tell the difference between the two. A person who has experienced it knows what he is talking about, and to the person who you are granting help will get better soon because they will relate to what you are talking about because they will have the mentality of saying “If my coach was able to walk the road, and yet he is alive and cannot be detected, I can also walk the road too.”* (013, HIV, 5 years, Eastern Cape)

Peer coaching appeared to open a different kind of relational space where vulnerability became more acceptable because another man with shared experience modeled it. Coaches often began the relationships by disclosing their own histories of TB or HIV to establish credibility and signal that talking about illness is permissible between men.

### Masculinities and Health Behavior

The results reflect hegemonic expectations of self-reliance and control intersecting with clinic environments to shape men’s health-related actions, including clinic avoidance, disclosure decisions, and treatment adherence. Coaches described how clinics are experienced as feminized or disempowering spaces that threaten masculine status, and how peer coaching works by reframing health-seeking as responsibility and strength rather than weakness.

#### Clinic Avoidance and Masculine Status

Coaches often identified masculine expectations around stoicism, self-reliance, and emotional restraint as primary barriers that prevent men from accessing health care services. Many coaches described how men avoid clinic attendance due to concerns about being perceived as weak or vulnerable. One coach noted that “many still believe that if you go to a clinic, you are weak,” and emphasized that such beliefs are “taught by other men,” reinforcing a collective norm that clinics are not for men (006, HIV, 5 years, Eastern Cape). Others described how men refuse to wait in queues or spend a day at the clinic because “that mind-set of ‘I’m a man, I can’t spend my day here’ affects the way they go about things” (003, TB/HIV, 11 months, Eastern Cape).

*That is a negative impact, my brother . . . Because when you’re stereotyped and you have that mentality that men are not allowed to seek help at the clinic, a man cannot do certain things. I think in terms of the percentage, maybe 90% of males are still stereotyped about coming to the clinic . . . I would include myself, my brother, because when I was diagnosed with TB, I was* [HIV] *negative and told myself that it will soon go away. If I could steam and vomit using traditional medicine from the traditional healer, I would be fine.* (001, TB/HIV, 11 months, Eastern Cape)

*Another issue is that at the clinic is that they [nurses] shout loud when they are calling a patient. As a result, you would be known by someone who didn’t even know that you are taking treatment from the clinic. And that decreases your confidence, because sometimes we don’t want to be seen by all the people that we are taking treatment.* (002, TB/HIV, 11 months, Eastern Cape)

Within the clinic flow, players described to the coaches how they were required to disclose personal health information to several non-clinic staff members, such as security guards and administrative personnel. This process heightened concerns about privacy, as it made it easy for others in the community to learn about a man’s clinic attendance and fostered fears that staff could easily gossip about patients’ health matters. One coach described how a patient must explain his condition multiple times, to the security guard, queue marshal, and receptionist, before seeing a nurse: “Before I even go see the nurse, four people already know what is wrong with me . . . as men, we are impatient we cannot wait a long time” (007, HIV, 3 years, KZN). These structural barriers intersected with masculine norms to reinforce care avoidance.

#### Reframing Adherence as Responsibility and Strength

Coaches described deliberately leveraging concepts of responsibility, family protection, and strength to motivate treatment adherence. They simultaneously address resistance by framing health-seeking and medication routines as acts of resilience and care for loved ones, aligning adherence with valued masculine roles. They described counseling men who initially refused treatment by appealing to their responsibilities: “you said you work for your family . . . if you do not take this treatment . . . who is going to take care of your baby?” (020, HIV, 1 year, Western Cape).

*He does not see himself as a person when he is taking treatment. He does not see himself as man enough. So, I played up to him the responsibility, I approached him to talk as a man to man, both of us, so that things would be okay. You do not have another life when you can do the thing we’re about to take. I told him that you do not live for me. You are living for yourself. Do not look at people, because people who love you are your family and your child. You must take the treatment.* (014, HIV, 5 years, Eastern Cape)

#### Creating Male-Centered Spaces and Flexible Pathways

Coaches also described seeing successful health behavior change among their players and acknowledged needing to work within existing masculine ideals rather than challenging them directly. Creating “Men’s Corners” and other masculinized health care spaces will reduce stigma and accommodate men’s preferences for efficiency and privacy. These dedicated areas in clinics are specifically designed to be male-friendly, providing an inviting atmosphere where men feel more comfortable accessing health services (Health-e News, 2019).

*In a Men’s Corner, the men would be free because sometimes they are afraid of being seen by people who know them from their community. I believe their attitudes are controllable when they are in the Men’s Corner. Otherwise, yes, men have attitudes towards seeking health because of being afraid of being seen by others. Some would go back [leave] while they are at the gate once they see the long queue in the clinic.* (004, HIV, 4 years, Eastern Cape)

Coaches implemented practical strategies, such as providing transportation support, offering flexible scheduling, delivering medication to men’s homes, arranging alternative collection times, and traveling long distances to reach players, to help men overcome logistical impediments and maintain their treatment. This was especially important when self-reliance might otherwise prevent clinic attendance. One coach described organizing food parcels when a player said, “I do want to take these pills but . . . I do not have food at home” (018, HIV, 5 years, Eastern Cape). By addressing these barriers, coaches directly engaged with and adapted to masculine norms and ideals around independence and reluctance to seek help, which often shape men’s health behaviors and treatment adherence.

*There was a day that I called and the player said “I know that today is my date to come to the clinic, but I could not come because i do not have money to come to the clinic . . .. I do not have food.” As a result, all of that did not sit well with me. I had a thought of what if it were me or my sibling in this situation. So, what I did was talk with a social worker who had a player in a similar situation, and he was given parcels from the clinic. We arranged to meet with him on a specific day. I, as the coach, told him that we can meet . . . I told him that when he does not have money, he should tell me before his date, because I will also be able to plan financially as the coach, because he is my player.* (005, HIV, 2 years, Eastern Cape)

### Masculinities and Physical Health

The ideals of masculine strength and denial intersect with structural conditions across the life course to shape men’s physical health. Coaches described that many men reinterpret serious symptoms as temporary disruptions to toughness rather than as signs requiring medical evaluation. They also note that poverty, food insecurity, and provider pressures further constrain men’s ability to prioritize their own health.

#### Symptom Denial

Coaches consistently observed how traditional ideals of strength and self-reliance directly compromised men’s engagement with TB and/or HIV care. Men’s reluctance to acknowledge vulnerability often manifests as symptom denial or delayed health-seeking. Coaches describe how many players dismissed early warning signs, such as persistent coughs or weight loss, as temporary inconveniences rather than serious medical concerns. One coach describes a player who said he was “*not having any health problems, and his body was still strong.”* (004, HIV, 4 years, Eastern Cape) When he eventually sought care, moments of emotional response, as a player moved to tears upon reconnecting with a supportive coach, highlight both the depth of their prior resistance and the significance of trusted relationships in reshaping attitudes toward their health.

*Men find stigma very hard. Stigma from the community. Stigma from the family. Even when they just walk into the facility. They already have that mentality, “I’m going to be judged. I’m going to be judged; I’m going to be judged.” And that makes men not even want to go to the facility. And they’d rather stay at home, and they will go to the facility when they are totally, totally sick.* (012, HIV, 8 months, Western Cape)

#### Provider Role, Work, and Treatment Demands

Beyond the core health challenges faced by participants, the intersection of poverty and food insecurity emerged as a significant barrier that worsened treatment tolerance. Coaches described how coaching was exacerbated by structural factors such as food insecurity and labor conditions. These not only intensified physical suffering but also undermined treatment efficacy (some people taking TB treatment require particular nutritional supplementation to avoid severe side effects).

*I can say it was hearing a story of a client who comes from an underprivileged background, where it is hard for him to take treatment because there is no food at home. He is not working, and no one is employed at home. Financially, the situation is bad. Most of the time, there is no food at home.* (017, HIV, 5 years, Eastern Cape)

Some coaches linked men’s health decisions directly to money and food insecurity. They described how men weighed a day at the clinic against a day’s wages, and how taking TB treatment without reliable access to food could feel unbearable. One coach noted that the expectation to be the breadwinner was used to argue against attending appointments, with family members asking why a man would “choose” the clinic over work. Together, these pressures led to putting off starting treatment, missing visits, and stopping medication, even when men knew what was at stake for their health.

*[My player] told me that “I do not get support at home. At home I have a wife, I live with my grandchildren and children. I do not get support from home. I have to go to work, and if I do not, then my wife will shout at me. And sometimes on those days I am supposed to come to the clinic, I say not today. I will not be able to go to work, I have to go to the clinic for my appointment. My wife would shout at me and say you are choosing to go to the clinic instead of going to work? What will we eat since we have children?”* (010, HIV, 1 year Western Cape)

### Masculinities and Mortality Risk

The results show that traditional masculine expectations and structural inequities combine, producing late presentation, treatment interruption, and elevated risk of premature death. Coaches described men’s unwillingness to acknowledge vulnerability, fear of judgment, and concerns about masculine status as contributing to delayed testing and engagement in TB/HIV care.

#### Stoicism, Judgment, and Late Presentation

Coaches frequently described how men’s refusal to acknowledge symptoms or seek timely medical intervention often resulted in advanced disease progression that increased the risk of death. Interviews provided rich text regarding how masculine expectations around stoicism and self-reliance create dangerous health behaviors, including delayed care-seeking and disengagement from care. One coach described a player who came “crying . . . feeling weak,” unable to eat, having avoided testing for nearly a year. When tested, he was HIV positive and severely ill, having gone from size 38 to size 28 clothing (005, HIV, 2 years, Eastern Cape). This pattern of enduring symptoms until crisis reflected the expectation that men should tolerate discomfort without complaint, directly increasing mortality risk. Coaches further described how culturally resonant peer coaching approaches that honor rather than challenge men’s sense of masculine identity would engender changes in health behaviors. Coaches also described that the expectation for men to endure discomfort without complaint led to advanced disease progression among their players. They suggest that clinic attendance and medication routines are expressions of resilience rather than weakness.

*I would say guys should not feel shy or afraid when having to go to the clinic. We are also human. As guys, you do get sick like other people, so we must not feel ashamed. At the end of the day, we must go for the sake of lives. As guys you can’t allow yourself to lose weight and become tiny while there are clinics made for all of us. I would say [a] man is somebody [who] would be confident about himself and not listen to what others are saying.* (002, TB/HIV for 11 months, Eastern Cape)

#### Risk Denial and Abandonment of Treatment

Coaches described how men’s denial of risk, often manifesting as downplaying of symptoms or delayed care-seeking and diagnosis, is tied to masculine risk denial.

*There is this guy who is a taxi driver, and he was diagnosed last year in October. When he came in, his condition was very bad. This goes back to what I was saying that males do not want to get tested. When he came in, he was in a bad condition. They end up coming to the clinic when they are vulnerable. That is where coaching begins. Once they get initiated, they start treatment because some, most of the guys resist taking treatment because of the stigma. That is when you explain to him as the coach that “I have also been through this journey and for you to have a good life you, you have to take your treatment and used condoms.”* (018, HIV, 5 years, Eastern Cape)

#### Peer Survivorship as Disruption of Fatalism

Coaches described how their own stories as “survivors” and visible health served as powerful counter-narratives to fatalistic beliefs about HIV and TB diagnosis. Their stories helped men understand that sustained engagement with treatment was not only compatible with masculine identity but essential for fulfilling masculine roles as providers and protectors. One coach observed that when players see that “this person, I as a coach, have gone through this, and I am still here,” it “gives them assurance that they are in good hands” and that they too can “live their life and conquer whatever they are going through” (012, HIV, 8 months, Western Cape). By sharing their story and offering ongoing support, coaches helped men reinterpret clinic attendance and medication routines as expressions of resilience rather than weakness. Redefining their outlook disrupted the harmful scripts that link acknowledgment of illness to loss of manhood and, ultimately, to preventable mortality.

*Ninety percent of players come to the clinic with signs that indicate their condition. They come when they can see that they do not have any other options because they believe “a man does not get sick” or “a man must not show his emotions.” So that is where, as a coach that is placed at the Men’s Health side, I need to play a big role and show him that this is one of the things that kill us as black people . . . Everyone is sick, but sicknesses are different. Can you see that you are making a spectacle of yourself by waiting for too long to come to the clinic?* (020, HIV, 1 year, Western Cape)

## Discussion

**Figure 1.**
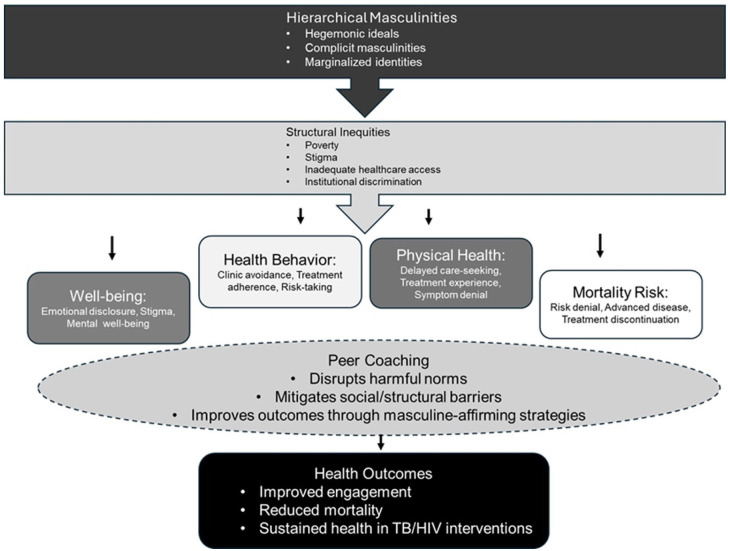
HIMM and Peer-Support Intervention.

Applied to this study, which explores how constructs of masculinity contribute to men’s health disparities, the HIMM framework provides a lens for understanding how Coach Mpilo disrupts harmful masculine norms while leveraging adaptive masculinities to improve health engagement (Evans et al., 2011). The framework illuminates how men’s avoidance of clinical care is often tied to hegemonic ideals of stoicism and self-reliance, and intersects with structural barriers like stigmatized clinic environments and work-related absences (Evans et al., 2011; Seidler et al., 2024). Peer coaches, often embodying relatable masculinities, bridge these gaps by reframing vulnerability as strength and advocating for gender-sensitive services like “Men’s Corners” (Lefkowich & Richardson, 2018; Seidler et al., 2024; Wippold et al., 2024). By addressing the intersection of hierarchical masculinities with social determinants (i.e., poverty, stigma, and inadequate health care systems), the HIMM framework helps to explain how the Coach Mpilo intervention works with, rather than against, masculine identities, thus supporting men to engage in positive health-promoting behaviors without compromising their sense of manhood while simultaneously addressing structural inequities that exacerbate health disparities (Evans et al., 2011; Medina-Marino et al., 2019; Oliffe, 2023). Figure 1 illustrates how four interrelated domains structure the main findings of our study on men’s health trajectories. Together, these domains map the ways difficult clinic procedures, masculine norms, and social expectations influence men’s engagement with care, emotional health, and treatment outcomes across different stages of illness and the care cascade. It advances understanding of why men may delay care, resist status sharing, and experience greater risk of disengagement and adverse health outcomes.

### Masculinities and Well-Being

The results provide key observations into how masculine norms profoundly contribute to men’s emotional well-being and subsequent health outcomes in the context of TB and HIV care. Traditional masculine norms around stoicism and emotional restraint deter emotional disclosure and help-seeking behaviors among men with TB and HIV (Chikovore et al., 2017; Daniels et al., 2021; Hooker et al., 2012). Coaches consistently identified emotional isolation, that “men cannot share issues with each other,” as a key factor undermining treatment adherence, with many men struggling to process their diagnosis or seek support due to fears of appearing vulnerable (Medina-Marino et al., 2025; Yousaf et al., 2015). This aligns with previous research showing that men who endorse traditional beliefs about manhood engage in poorer health-related behaviors and face greater health risks than men who hold less traditional beliefs (Caperchione & Sharp, 2025; Ojedokun & Adebayo, 2025).

Coach Mpilo emerges as a transformative intervention that directly challenges harmful masculine norms by creating safe spaces where emotional expression is not only accepted but actively encouraged (Sharma et al., 2017). By sharing their own lived experiences with TB and/or HIV, coaches demonstrate that vulnerability can be reframed as strength rather than weakness, helping men develop new understandings of masculinity that incorporate emotional openness and help-seeking as compatible with masculine identity (Evans et al., 2011). These interactions suggest that peer coaching works partly by creating a permission structure for emotional expression. Disclosure is then reinterpreted as courage rather than weakness, aligning with HIMM’s view of masculinities as dynamic and relational. This approach aligns with gender-transformative interventions aiming to reduce inequality by challenging harmful gender norms, promoting positive masculine patterns, and promoting more equal systemic power relationships (Ruane-McAteer et al., 2019).

### Masculinities and Health Behavior

The findings reveal how masculine norms shape men’s health-seeking behaviors and engagement. Consistent with the HIMM framework, our findings demonstrate that men’s health care avoidance is often rooted in hegemonic masculine ideals that position help-seeking as a sign of weakness (Evans et al., 2011; Oliffe, 2023). Coaches consistently identified specific structural and behavioral patterns that create barriers to care, including reluctance to wait in clinic queues, resistance to being seen at health care facilities, and breaches of confidentiality with clinic personnel. Having to disclose personal health details multiple times to non-clinic staff, including security guards and administrative workers, heightened concerns about privacy and confidentiality, exposing men’s clinic visits to community scrutiny and gossip. Such fears reinforced men’s reluctance to seek care, underlining the need for more private, male-sensitive clinic processes to reduce stigma and improve health care engagement. These findings align with research showing that health-promoting behaviors are often associated with femininity while health-harming behaviors are linked with masculinity, contributing to disparities in health outcomes between men and women (Griffith et al., 2016; Sharma et al., 2017; Thorpe et al., 2016).

The peer coaching model addresses these barriers by reframing health-seeking behaviors within culturally acceptable masculine ideals (Hooker et al., 2012; Maga et al., 2025; Mashilo et al., 2025; Novak et al., 2019). Coaches leverage concepts of responsibility, family protection, and strength to motivate treatment adherence, aligning health care engagement with valued masculine roles as breadwinners and family leaders. This approach resonates with the HIMM framework’s emphasis on masculine-affirming health interventions that work with rather than against men’s gender identities (Evans et al., 2011). By positioning treatment adherence as an act of responsibility to family and community, coaches help men reconcile health-promoting behaviors with their masculine identities. The creation of “Men’s Corners” and other masculinized health care spaces may reduce stigma and accommodate the need for efficiency and privacy. This aligns with research showing that gender-transformative approaches are more effective when they provide men opportunities to reflect on dynamic, often conflicting images of masculinity and empower them to renegotiate masculine norms in ways that support getting care. The Coach Mpilo intervention demonstrates how peer support can create bridges between traditional masculine ideals and health-promoting behaviors, particularly when delivered by men who embody both masculine credibility and positive health outcomes (Oliffe, 2023; Sharma et al., 2017).

### Masculinities and Physical Health

The intersection of masculine norms and physical health reveals how traditional ideals of strength and self-reliance compromise men’s health. Our results demonstrate how hegemonic masculine norms create patterns of health denial whereby men reframe significant clinical symptoms as temporary disruptions to their physical strength rather than acknowledging them as indicators requiring medical evaluation. This pattern aligns with research showing that men often prefer to risk their physical health rather than be associated with traits they perceive as feminine, such as vulnerability or weakness (Chikovore et al., 2017, 2020; Daniels et al., 2021).

Beyond individual behaviors, our findings highlight how structural factors (i.e., food insecurity; the inability to take time from work to seek care) intersect with provider roles associated with manhood to create unique vulnerabilities. Poverty forces men to choose between clinic attendance and income generation, while TB treatment demands consistent nutrition, which is often inaccessible. The expectations that men must provide for others before addressing their own health needs, “My wife would shout at me and say you are choosing to go to the clinic instead of going to work,” illustrates these negative masculine norms are just very often forced on men. These intersecting factors have profound consequences for men’s health, contributing to late presentation, poor treatment adherence, and increased risk of severe illness or death. Some coaches described players who disengaged from care or were slow to start treatment due to these challenges, underlining the need for interventions that address both social norms and structural barriers to improve health outcomes for men. The coaches in our study describe how they address these issues through transportation support, flexible scheduling of peer-support sessions, medication delivery, and home visits. This dual approach, which addresses both individual behaviors and systemic constraints, builds trust and creates viable care pathways.

### Masculinities and Mortality

The relationship between masculine norms and mortality risk emerges as one of the most consequential domains in our analysis. The HIMM framework helps us understand how the social strictures that perpetuate traditional masculine ideals directly contribute to life-threatening health outcomes among men with TB and HIV. Specifically, coaches describe how many men are not equipped with the skills to overcome perceptions of health-related weaknesses as a threat to their manhood, resulting in delayed health-seeking and increased mortality risk. Coaches can uniquely describe the first-person experience of fear, observe men’s perception of their own mortality, and the exacerbation of these issues as members of the health care system. They are key informants in the health behavior cycle. These patterns align with epidemiological evidence showing that men in South Africa are disproportionately affected by TB, accounting for over 60% of microbiologically confirmed pulmonary TB cases while representing a smaller proportion of those who successfully complete treatment (Chikovore et al., 2020; Medina-Marino et al., 2022).

The Coach Mpilo intervention addresses these mortality risks by disrupting the masculine narrative that equates medical compliance with weakness. Coaches’ lived experiences of surviving TB and HIV offer alternative models of masculinity that directly challenge fatalistic narratives, illustrating that actively managing one’s health can affirm traditional roles as providers and protectors. By sharing their own visible health and resilience, coaches reframe treatment adherence as a legitimate expression of masculine strength and responsibility. When harmful masculine expectations remain unchallenged, they create a sequential progression from initial diagnosis to early mortality that peer-support initiatives can successfully intercept. Peer interventions are generally more effective when they involve sustained engagement, as ongoing support, regular follow-up, and relationship-building help individuals overcome challenges to treatment adherence and foster long-term positive health behaviors (Iwelunmor et al., 2016).

## Limitations

Some limitations should be considered when interpreting the findings of this study. The study’s planned sample size was reduced due to funding cuts from PEPFAR and USAID in early 2025, potentially limiting the diversity of experiences and perspectives captured across different geographic regions and socioeconomic contexts. The study exclusively interviewed coaches, and future research should apply the same research questions to the perspectives of the players. The cross-sectional nature provides only a snapshot, and future studies should capture how masculine identities and health behaviors evolve over time through sustained coaching relationships. The reliance on self-reported data from coaches introduces the potential for social desirability bias, given their professional investment in the program’s success. With some non-Xhosa English-speaking researchers investigating the experiences of South African men, positionality must be acknowledged. Our identity presents constraints that may limit comprehension of their experiences. To mitigate these limitations, the researchers engaged in community validation with the South African native isiXhosa-speaking research team and community members to confirm language use, translations, and contextual understanding of the data.

## Conclusions and Implications for Practice and Research

The Coach Mpilo intervention offers valuable insights for developing gender-transformative health interventions that effectively engage men in TB and HIV care. Our findings suggest that successful interventions should incorporate four key characteristics identified in the literature: multicomponent activities that include education, persuasion, modeling, and enablement; multilevel programming that mobilizes the broader community; and delivery by trained facilitators over a sufficient duration (Iwelunmor et al., 2016; Ruane-McAteer et al., 2020). The peer coaching model exemplifies these characteristics, providing a promising approach for addressing the complex interplay between masculinity and health behaviors.

To improve Coach Mpilo uptake, the program could be strengthened by providing additional resources for food and transportation support, as both coaches and participants frequently cited food insecurity and travel hurdles as major obstacles to treatment adherence. Future research should focus on developing theory-informed interventions that explicitly address masculine norms across the full spectrum of TB and HIV services. While our findings provide valuable insights into how peer coaching can improve men’s engagement with testing and treatment initiation, more research is needed on interventions that support long-term treatment adherence and retention in care. In addition, interventions should be tailored to address the specific needs of diverse groups of men, recognizing that masculinity intersects with other social identities to create unique health experiences and vulnerabilities.
